# Growth of Hexagonal Columnar Nanograin Structured SiC Thin Films on Silicon Substrates with Graphene–Graphitic Carbon Nanoflakes Templates from Solid Carbon Sources

**DOI:** 10.3390/ma6041543

**Published:** 2013-04-16

**Authors:** Xingfang Liu, Guosheng Sun, Bin Liu, Guoguo Yan, Min Guan, Yang Zhang, Feng Zhang, Yu Chen, Lin Dong, Liu Zheng, Shengbei Liu, Lixin Tian, Lei Wang, Wanshun Zhao, Yiping Zeng

**Affiliations:** 1 Key Laboratory of Semiconductor Materials Science, Institute of Semiconductors, Chinese Academy of Sciences, Beijing 100083, China; E-Mails: gshsun@red.semi.ac.cn (G.S.); liubin2010@semi.ac.cn (B.L.); ggyan@semi.ac.cn (G.Y.); guanmin@red.semi.ac.cn (M.G.); zhang_yang@mail.semi.ac.cn (Y.Z.); fzhang@semi.ac.cn (F.Z.); donglin09@semi.ac.cn (L.D.); liuero@semi.ac.cn (L.Z.); liushengbei@semi.ac.cn (S.L.); tianlixin@semi.ac.cn (L.T.); wangl@semi.ac.cn (L.W.); zwshuke@semi.ac.cn (W.Z.); ypzeng@red.semi.ac.cn (Y.Z.); 2 Semiconductor Lighting Technology Research and Development Center, Institute of Semiconductors, Chinese Academy of Sciences, Beijing 100083, China; E-Mail: chenyu@semi.ac.cn

**Keywords:** hexagonal columnar, silicon carbide, thin film, graphene, nanoflake, solid carbon, CVD

## Abstract

We report a new method for growing hexagonal columnar nanograin structured silicon carbide (SiC) thin films on silicon substrates by using graphene–graphitic carbon nanoflakes (GGNs) templates from solid carbon sources. The growth was carried out in a conventional low pressure chemical vapor deposition system (LPCVD). The GGNs are small plates with lateral sizes of around 100 nm and overlap each other, and are made up of nanosized multilayer graphene and graphitic carbon matrix (GCM). Long and straight SiC nanograins with hexagonal shapes, and with lateral sizes of around 200–400 nm are synthesized on the GGNs, which form compact SiC thin films.

## 1. Introduction

Silicon carbide (SiC) is a kind of wideband semiconductor material and has numerous excellent properties, including high electron mobility, high breakdown voltage and high temperature endurance [1,2], which make SiC one of the most attractive electronic materials nowadays because of its potential applications in high power, high temperature electronics [3]. SiC is polytypic; cubic SiC (3C-SiC) and hexagonal SiC (4H-SiC, 6H-SiC) are the most common materials among its polytypes. Both 4H-SiC and 6H-SiC materials are industrial mature and bulk crystals which can be mass produced by physical vapor transport method (PVT) above 2000 °C [4,5], and their corresponding homoepitaxial thin films of n-type and p-type can be grown by chemical vapor deposition (CVD) [6,7]. Power devices based on these hexagonal SiC polytypes have already been commercialized [8,9]. However, applications based on homoepitaxial hexagonal SiC still suffer from the high process cost issue due to the high energy consumption and the low yield of the PVT. If hexagonal SiC films can be heterogrown on silicon substrates, it will bring significant benefits. However, cubic SiC is often the most common polytype deposited on silicon substrates [10,11]. Although carrier mobility of 3C-SiC is higher than that of hexagonal SiC, the bandgap of cubic SiC is smaller than that of hexagonal SiC [12]. In addition, the high carrier mobility of 3C-SiC is hindered by the imperfect crystalline SiC grown on silicon substrates due to the large lattice mismatch and the thermal mismatch between silicon and 3C-SiC [13].

Recently, graphene, a plane layer of graphite, with hexagonally arranged carbon atoms, emerges as a new material with fantastic physical and chemical properties, and shows potential applications in both semiconductor electronics [14,15] and templates for material synthesis [16,17,18,19]. Since the planar structure of graphene is similar to the C plane of crystal with a hexagonal crystalline structure, it is straightforward to utilize graphene as a template for hexagonal crystal growth on hetero substrates. GaN growth on graphene template with ZnO nanowalls or with low temperature GaN (LT-GaN) as an intermediate layer has already been demonstrated, and good results have been achieved [16,17].

The common homoepitaxial growth of hexagonal SiC adopts step-control mechanism to avoid polytypic inclusions by using off-axis substrates [20], and the heteroepitaxial growth of cubic SiC on Si substrates adopts carbonized Si as buffer layer [11,13]. So far, graphene as buffer layer for SiC growth on insulator structures has also been reported [21]. The situation of carbon template for SiC is different from that of GaN or ZnO; carbon is one chemical component of SiC. In the processing of SiC synthesis on the carbon template, the carbon atoms will also serve as the carbon source therefore incorporated in the synthesized SiC. Thus the carbon template will be consumed and the template effect will become weaker or even worse, will vanish, especially if the carbon template is of one layer thickness. In fear of this, in this work, we use a method to feed carbon templates continuously to the growth frontier during SiC growth. We notice that graphene–graphitic nanoflakes (GGNs) will exfoliate from carbon foam at high temperature under H_2_ ambience. It is feasible to transport carbon templates continuously to the growth frontier when carbon foam is placed on the up-stream side of the Si substrate in the growth tube.

Therefore, this work is aimed at investigating the applicability of GGNs as carbon templates for the growth of SiC thin films on Si substrates. Herein, by characterizing the surface and the cross-sectional morphologies, and the polytype of the synthesized SiC, it is found that the SiC thin films consist of hexagonal columnar nanograin structures.

## 2. Experimental Section

**Figure 1:**
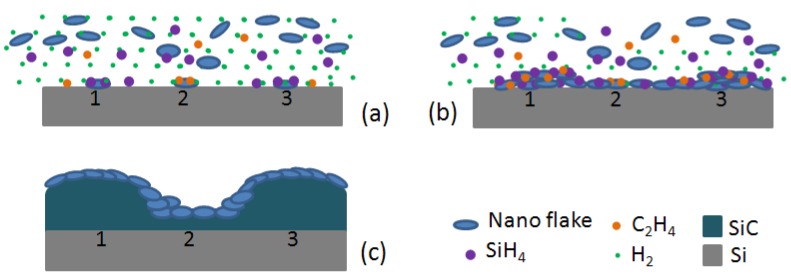
Deposition process illustrations.

The growth of hexagonal columnar nanograin structured SiC thin films was carried out in a custom-built, horizontally oriented low pressure chemical vapor deposition system. The growth chamber consisted of a quartz tube (70 mm inner diameter and 120 mm in length) with a 60 mm long hot-wall zone. The substrate temperature was monitored by an infrared thermometer, and the chamber pressure was controlled by a stack valve. Before growth, the 2-inch Si(100) substrate was carefully treated with surface contamination cleaned by a RCA processing and native oxides removed by a 30% diluted HF solution bathe for 30 s, then dried under N_2_ flux. High pure carbon foam (purchased from Beijing Sanye Carbon Co., Ltd., Beijing, China, Purity > 99.999%) was used as the solid carbon source for the preparation of graphene–graphitic carbon nanoflakes (GGNs). The carbon foam was placed at the up-stream side of the Si substrate in the hot-wall zone of the tube. At a high temperature of more than 500 °C, GGNs was exfoliated from the carbon foam by H_2_ intercalation, and was continuously fed to the surface of the substrate. The silicon and the carbon precursor were SiH_4_ and C_2_H_4_, respectively, and the purified H_2_ was used as the diluted gas and the carry gas for the precursors. The growth process lasted 60 min at approximately 40 Torr with the substrate temperature of 1120 °C. Several growth processes have been conducted, and the uniformity of the growth process was better than 99%. Figure 1 showed the deposition process. Firstly, GGNs were fed to the surface of the substrate together with silicon precursor (SiH_4_) and carbon precursor (C_2_H_4_) diluted in H_2_; GGNs reacted with absorbed precursors were deposited on substrate surface as nucleation centers. Secondly, more nucleation centers formed after a deposition period; substrate surface was covered by continuous SiC thin film. Lastly, after growth process, the remain GGNs in the chamber were deposited on the SiC film surface after precursors were turned off.

The surface morphologies and structures of the sample were investigated by a Normaski microscope (Olympus Inc, Tokyo, Japan) and a Hitachi S-4800 field-emission SEM (Hitachi Inc, Tokyo, Japan) operated at 15 kV. The phases structures of the sample were identified by the X-ray diffraction (XRD) patterns using a X’Pert-PRO MPD (PANalytical Inc, Almelo, the Netherlands) with Cu K_α_ (λ = 0.15406 nm). The cross-sectional structures were characterized by TEM investigations, which were carried out using a Tecnai G2 F20 U-TWIN (FEI Inc, Hillsboro, OR, USA) operated at 200 kV. Conventional bright field TEM images and selective area electric diffractions (SAED) pattern were used to identify the nanograin structures of the SiC films. Raman scattering measurements were performed by a Jobin-Yvon HR 800 (Horiba Jobin Yvon Inc, Paris, France) instrument in a backscattering geometry, with a solid-state laser source of 632.8 nm wavelength in a macroscopic configuration.

## 3. Results and Discussion

**Figure 2:**
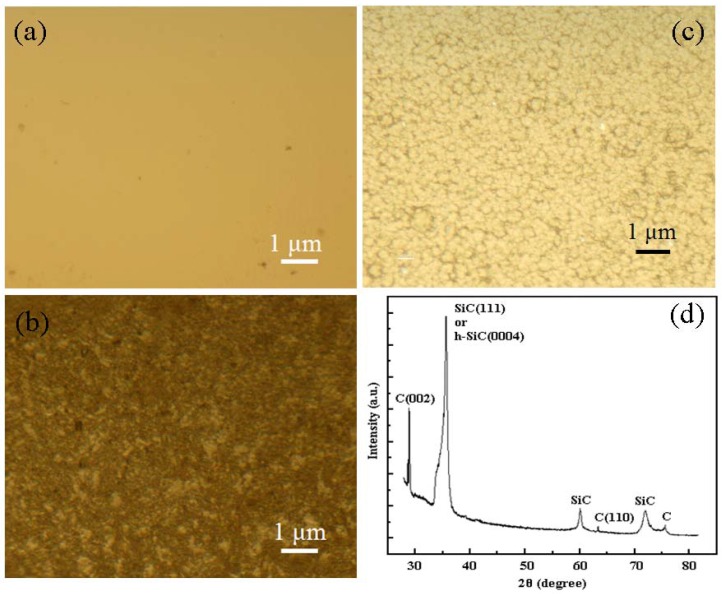
Morphologies of: (**a**) as-received substrate surface; (**b**) GGNs covered surface; (**c**) SiC film surface; and (**d**) XRD patterns of the thin film after SiC deposition.

The surface morphologies of the samples are observed via a Normaski microscope. Figure 2 shows their morphologies. The Si substrate is very clear and smooth, without any observable features. When covered with GGNs, the Si surface is rough and colored light black. The surface of the deposited SiC film is grainy, with a feature size of several hundreds nm. XRD spectrum is used for material characterization after film deposition. Several peaks are observed in Figure 2d, and can be identified as signals of SiC and carbon. This indicates that SiC has been synthesized during the growth process. The SiC film is polycrystalline. The carbon in the deposited SiC thin film is the remaining GGNs.

**Figure 3:**
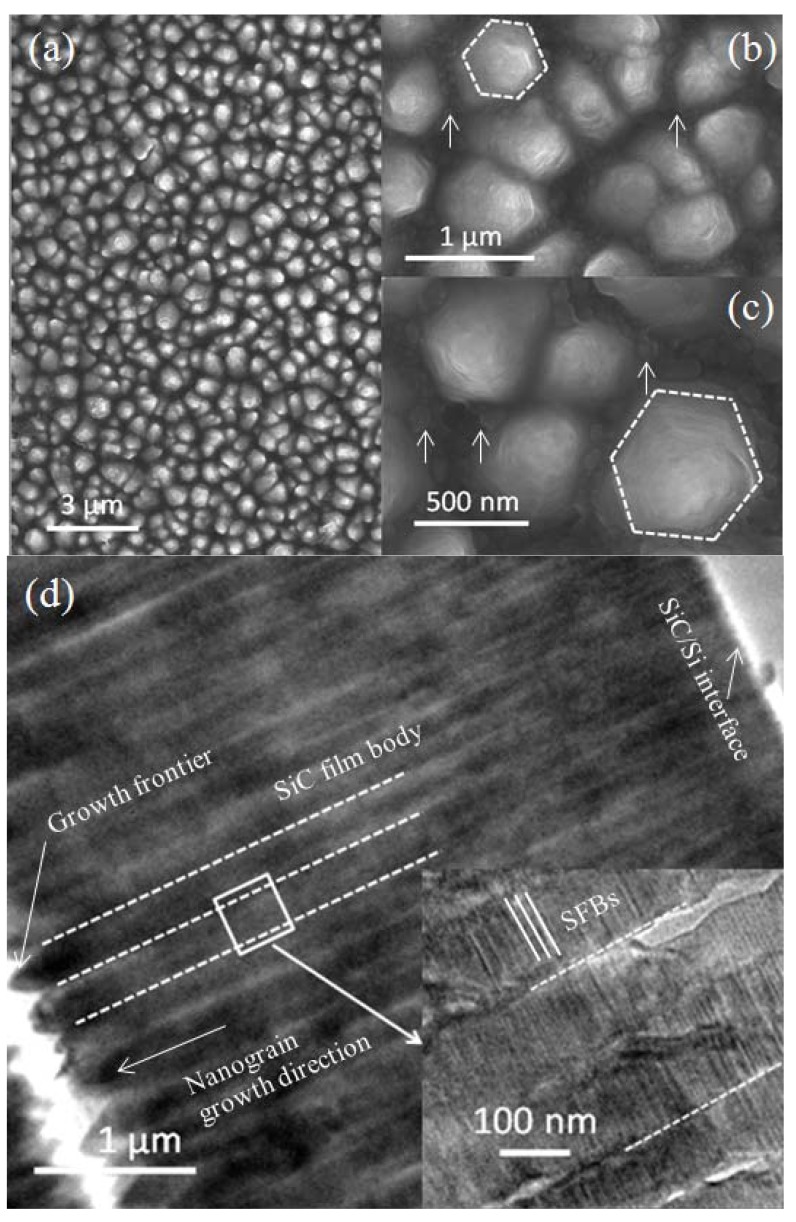
**(a)** Field-emission scanning electron microscopy (FESEM) image of surface morphologies of SiC films; **(b,c)** magnified images of (a). The dotted white hexagonal shapes are guides to the eye, and the white arrows in (**b**) and (**c**) point to spacers and GGNs between SiC nanograins, respectively; **(d)** TEM image of cross-sectional morphologies of SiC films. The right-bottom corner of (**d**) shows a magnified image of the nanograins. The parallel dotted white lines are guides to the eye.

The surface morphologies and structures of the synthesized SiC thin films are further investigated by field-emission scanning electron microscopy (FESEM). Generally, the samples consist of nanograins (Figure 3a). Figure 3b,c show magnified images of SiC surface morphologies. It is clear to observe that these nanograins have hexagonal shapes, and that the side length of the nanograins are around 200–400 nm. There are spacers between the nanograins (see white arrows in Figure 3b), which indicates that nanograins do not coalesce to each other on the topmost surfaces (or the growth frontier). In the magnified image (Figure 3c), it is found that the spacers are filled with GGNs. The GGNs are small plates with lateral sizes of around 100 nm and overlap with each other (see white arrows in Figure 3c).

Figure 3d shows the transmission electron microscopy (TEM) image of cross-sectional morphologies of SiC films. The columnar features of the nano crystallites are clearly displayed in Figure 3d. The crystallites are straight, without branches to each other, crossing through the whole film, and are about 5 μm in length. Although the crystallites are separated at the growth frontier, they closely contact each other from SiC/Si interface to film body. In the magnified image (Figure 3d insert), more details are displayed. The lateral sizes of the crystallites are about 300 nm, in the range of the sizes of the side length of the nanograins, 200–400 nm, as can be seen in in Figure 3a. Stacking faults bundles (SFBs) have uniform distribution among the nanograins, with their normal directions parallel to the SiC nanograin growth direction.

**Figure 4:**
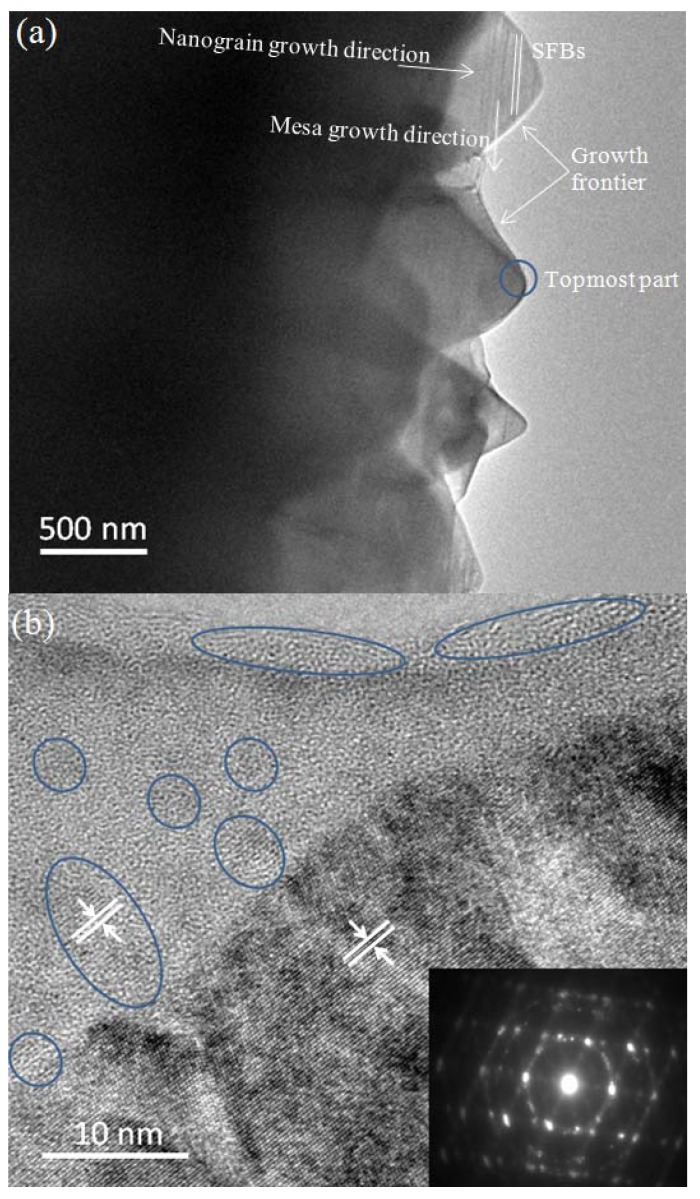
**(a)** TEM image of SiC films in cross section near the growth frontier; **(b)** HRTEM image of the topmost part of an individual SiC nanograin (blue circle in (a)) and its corresponding selective area electric diffractions (SAED) pattern. The blue ellipses indicate nanosized multilayer graphene.

The microstructural properties of SiC thin films near the growth frontier are further investigated by cross sectional-view bright field (BF) TEM image and selected area electron diffraction (SAED) pattern (Figure 4a,b). Although in the plane view SEM image (Figure 3a–c) the topmost parts of nanograins have hexagonal shapes, in the cross sectional-view TEM image they show triangular shapes. SFBs also clearly appear as can be seen in the insert on Figure 3d. When comparing Figure 3b,c and Figure 4a, we could deduce that SiC crystalline planes between SFBs form mesas. These mesas have hexagonal shapes in-plane (Figure 3b,c), and they have various sizes. Judging from the growth frontier (Figure 4a), the mesas’ sizes become smaller along the nanograin growth direction, thus the topmost parts of nanograins show triangular shapes in the cross sectional-view TEM image (Figure 4a). However, the mesas’ sizes of an individual nanograin in the film body are almost uniform (Figure 3d). The nanograin growth direction is perpendicular to the mesa growth direction. The growth rate of mesas of each individual nanograin are almost the same, thus the triangular shaped growth frontiers and the uniform dimensions of the individual nanograins in the film body remain during the whole growth process.

Figure 4b shows the high resolution TEM (HRTEM) image of the topmost part of an individual SiC nanograin. The SiC mesas are fully covered by GGNs. There are regular nanosized multilayer graphenes embedded in the graphitic carbon matrix (GCM) and the measured carbon plane spacing is 0.35 nm. Continuous multilayer graphene belt is observed on the outmost side of the GCM. The SiC planes with a measured plane spacing of 0.43 nm are parallel to the carbon plane of graphene. This indicates that the template effect of GGNs to SiC growth is obvious. The SAED pattern (Figure 4b insert) shows clear six-fold symmetry, indicating single crystalline hexagonal structure in this region [22,23]. Since SFBs are observed in the TEM image in Figure 3d insert and Figure 4a, the SAED also displays a ring pattern [24].

**Figure 5:**
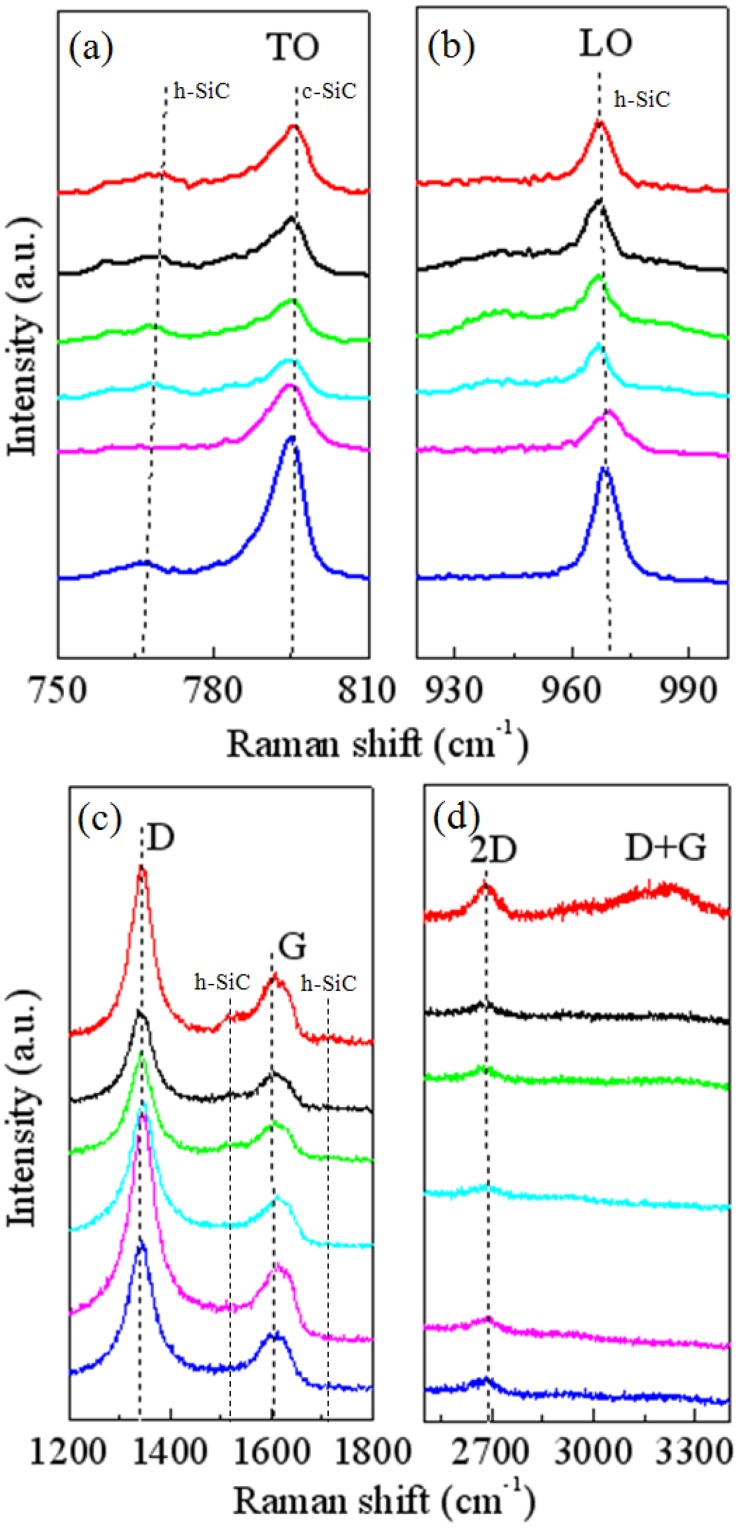
Raman spectra measured at six different points on our sample. **(a)** TO bands; and **(b)** LO bands of Raman spectra of SiC films; **(c)** D bands and G bands of Raman spectra of GGNs, together with second-order SiC bands; **(d)** 2D bands of Raman spectra of GGNs.

Raman scattering spectroscopy is of an extremely sensitive, non-destructive, and non-invasive characterization technique. It is often used as fingerprint identification to investigate SiC polytypes and crystalline qualities. Figure 5 shows the Raman spectra measured at six different points on our sample.

The main features in Figure 5a,b are the obvious TO bands located at 795 cm^−1^ and the LO bands located at 965 cm^−1^. Weak TO bands located at 766–770 cm^−1^ also appear in Figure 5a. The obvious TO bands can be identified as the fingerprint of cubic SiC (c-SiC) while the LO bands and the weak TO bands as that of hexagonal SiC (h-SiC) [25]. These bands are asymmetrical, indicating the SiC films are polycrystalline. The peaks positions and the frequency-integrated intensities vary slightly according to the six sampling points. Since the sampling points are randomly selected on our sample film, we can assess that the uniformity of the obtained SiC thin film is good across the whole wafer. Second-order Raman scattering peaks at ~1520 cm^−1^ and ~1717 cm^−1^ are also observed in Figure 5c alongside the D bands and G bands of GGNs. They are h-SiC related peaks, which indicate the hexagonality property of the SiC films.

GGNs at the growth frontier still remain unfolded. We investigate GGNs by Raman spectra. Primary features of GGNs including the defect induced D bands at ~1343 cm^−1^, the in-plane vibrational G bands at ~1608 cm^−1^, and the two-phonon 2D bands at ~2683 cm^−1^ are shown in Figure 5c,d, respectively. The peaks of D bands, G bands, and 2D bands of the six spectra are at the same position, that of resistivity. The D band is thought to be mostly the result of structural disorder and defects such as domain boundaries [26,27,28]. The high intensities of D bands indicate that the nanosized multilayer graphenes distribute randomly in the GCM. The ratio of the frequency-integrated intensities of D bands (*I_D_*) over G bands (*I_G_*) is greater than 1, which is the defective signal of graphene [29,30]. Since GGNs are made up of nanosized multilayer graphene and graphitic carbon matrix, this phenomenon is reasonable. The intensity of 2D band of one sample point (red line in Figure 5d) is higher than that of others points, which is attributed to the higher quality of embedded graphene at that point than others. A D+G band located at ~3231 cm^−1^ also appears in the Raman spectrum of that point, which indicates that the carbon matrix around that point probably is damaged since the D + G band reflect defects [31].

## 4. Conclusions

In conclusion, we have demonstrated the growth of SiC thin films with hexagonal columnar nanograin microstructures on Si substrates by using graphene–graphitic carbon nanoflake templates. The carbon templates are prepared via *in situ* H_2_ etching of high purity carbon foams on the up-stream side of Si substrates in the growth tube. TEM images and Raman spectra reveal that the carbon templates consist of nanosized multilayer graphenes which are embedded in the graphitic carbon matrix. SiC thin films grown on Si substrates by conventional low pressure chemical vapor deposition have been obtained, with their microstructures consisting of hexagonal columnar SiC nanograins with the assistance of carbon templates. The above results indicate that graphene–graphitic carbon nanoflakes can be used as potential templates for SiC thin film growth.
